# *Staphylococcus epidermidis* Urinary Tract Infection in an Infant

**DOI:** 10.1155/2012/983153

**Published:** 2012-08-09

**Authors:** Shankar Upadhyayula, Mamatha Kambalapalli, Basim I. Asmar

**Affiliations:** ^1^Department of Pediatrics, Children's Hospital of Michigan, Detroit Medical Center, Wayne State University School of Medicine, Detroit, MI 48201, USA; ^2^Division of Infectious Diseases, Children's Hospital of Michigan, Detroit Medical Center, Wayne State University School of Medicine, Detroit, MI 48201, USA

## Abstract

We describe the case of a previously healthy 7-month-old male infant with urinary tract infection due to *Staphylococcus epidermidis* grown from two separate urine cultures. Further evaluation showed severe bilateral vesicoureteral reflux. Physicians should not assume that *S. epidermidis* is always a contaminant in urine cultures.

## 1. Introduction

The majority of urinary tract infections (UTI) in children are caused by gram-negative coliform bacteria [1]. Gram-positive cocci, including enterococci, group B *Streptococcus,* and *Staphylococcus saprophyticus* have also been described as urinary pathogens [2–5]. 

*S. saprophyticus* infections are more commonly seen in young women and elderly men. *Staphylococcus epidermidis *urinary tract infection in healthy pediatric patients is rare and only a few cases have been reported in preadolescent children. 

*S. epidermidis* when isolated from the urine of previously healthy infants is almost always considered a contaminant. We describe the case of an infant with *S. epidermidis* isolated on two occasions from the urine during the same episode of illness. Further evaluation revealed severe bilateral vesicoureteral reflux.

## 2. Case Report

A previously healthy 7-month-old infant presented with fever for 2 days. He had runny nose for a week. Nasal wash for respiratory syncitial virus, influenza A, and influenza B antigens was negative. Urinalysis showed <5 white blood cells (WBC) per high power field (hpf). A diagnosis of viral illness was made and he was sent home. Urine culture (catheter specimen) subsequently grew 10^3^–10^5^ colony forming units (CFU)/mL of *S. epidermidis*. No treatment was given as the organism was considered a contaminant.

His fever persisted and he developed intermittent vomiting for 4 days. On admission, his temperature was 40.6°C; he was alert and in no distress. He was noted to be circumcised. Otherwise, physical exam was normal.

Laboratory data showed a white blood cell count (WBC) of 15,600/mm^3^ (70% neutrophils, 2% bands, and 17% lymphocytes). C-reactive protein (CRP) was 238 mg/L (normal <10 mg/L). Urinalysis showed 5–10 WBC/hpf and a catheterized urine sample was sent for culture. His electrolytes and renal function tests were normal. 

He was started on intravenous ceftriaxone treatment. He continued to spike fevers over the next 48 hours. Cerebrospinal fluid (CSF) showed no evidence of meningitis. Direct fluorescent antibody testing of respiratory secretions was negative for adenovirus and parainfluenza. Urine culture showed pure growth of *S*. *epidermidis,* >10^5^ CFU/mL. The organism was sensitive to vancomycin, trimethoprim-sulfamethoxazole (TMP-SMX), and gentamicin but, was resistant to ceftriaxone. His treatment was changed to intravenous TMP-SMX. Blood and CSF cultures were negative.

An ultrasound scan of the kidneys showed mild distention of the right urinary collecting system and mild prominence of the left pelvicalyceal system. The bladder was distended and contained multiple scattered areas of internal echoes. A vesico cystourethrogram (VCUG) revealed bilateral vesicoureteral reflux, grade 5 on the right and grade 4 on the left.

His fever resolved 36 hours after changing the antibiotic treatment. He was discharged to complete 10 more days of oral TMP-SMX treatment. Subsequently, he was maintained on prophylactic daily oral TMP-SMX. (He has done well and was followed by the urology service.)

## 3. Discussion 

*S. epidermidis* commonly causes infections associated with indwelling central venous catheters, cerebrospinal fluid shunts, prosthetic heart valves, and peritoneal dialysis catheters. When *S. epidermidis *is isolated from blood or body fluids in patients without predisposing factors, it is often considered a contaminant. Urinary tract infections caused by *S. epidermidis* are often associated with instrumentation of the urinary tract in a hospital setting, including neonates in the neonatal intensive care unit. 

Our patient was a previously healthy infant who presented with persistent fever and had elevated WBC and CRP. Urinalysis showed <5 WBC/hpf; however, his urine culture grew* S. epidermidis* on two occasions. He improved after appropriate antibiotic treatment. If *S. epidermidis* in the urine culture was considered a contaminant, the underlying vesicoureteral reflux would not have been identified.

A literature review (1980 to present) revealed 3 case reports of *S. epidermidis* UTI in children. All three patients were preadolescents. In one report by McDonald and Lohr [6], an 11-yr-old boy presented with fever, vomiting, and abdominal pain. Urine culture grew >10^5^ CFU/mL of *S. epidermidis* on two separate occasions. Imaging studies revealed smaller right kidney with evidence of cortical thinning and right-sided vesicoureteral reflux. In a second report, Hall and Snitzer [7] described two children (6 yr and 7 yr old) who presented with fever and shaking chills. Urine culture from each of these patients grew *S. epidermidis* on two occasions. Imaging studies identified vesicoureteral reflux in both cases. It is noteworthy that in both of the forementioned reports of *S. epidermidis* UTI there was lack of significant pyuria [6, 7]. This was also noted in our patient where urine microscopy showed only 5–10 WBC/hpf. This finding and the absence of urine nitrites, which are predominantly produced by gram negative bacteria, indicate that urine dip and microscopy are less helpful in diagnosing urinary tract infections due to *S. epidermidis. *Previous studies have suggested that in view of the tendency of *Staphylococcus *to form clusters, a count of 10^3^ CFU/mL may be a significant marker for UTI [5]. 

Urinary radiographic imaging in the previous two reports revealed underlying abnormalities in all 3 cases (vesicoureteral reflux). Our patient also has severe bilateral reflux. 

## 4. Conclusion

To our knowledge there are no published reports of similar cases in infants. In our patient if the *S. epidermidis* isolated from urine was considered a contaminant the underlying urinary abnormalities could have been missed. Isolation of *S. epidermidis* from urine (>10^3^/CFU) in a febrile infant, must be seriously considered as a pathogen even in the absence of pyuria. 

## Abbreviations

CFU: Colony forming units
WBC: White blood cells
HPF: High power field
CSF: Cerebrospinal fluid
VCUG: Vesico cystourethrogram
UTI: Urinary tract infection.

## References

[1] Lohr JA, Downs SM, Schlager TA, Long SS, Pickering LK, Prober CG (2008). . Urinary tract infections. *Principles and Practice of Pediatric Infectious Diseases*.

[2] Baciulis V, Eitutiene G, Cerkauskiene R, Baciuliene E, Jankauskiene A, Mieliauskaite E (2003). Long-term Cefadroxil prophylaxis in children with recurrent urinary tract infections. *Medicina*.

[3] Marosvári I, Kéry V (1990). Urinary tract infections caused by *Staphylococcus saprophiticus* in children. *Orvosi Hetilap*.

[4] Schille R, Sierig G, Spencker FB, Handrick W (2000). Urinary tract infections due to *Staphylococcus saprophyticus* in a child. *Klinische Padiatrie*.

[5] Abrahamsson K, Hansson S, Jodal U, Lincoln K (1993). *Staphylococcus saprophyticus* urinary tract infections in children. *European Journal of Pediatrics*.

[6] McDonald JA, Lohr JA (1994). *Staphylococcus epidermidis* pyelonephritis in a previously healthy child. *Pediatric Infectious Disease Journal*.

[7] Hall DE, Snitzer JA (1994). *Staphylococcus epidermidis* as a cause of urinary tract infections in children. *Journal of Pediatrics*.

